# Waterpipe-specific pictorial health warning labels are effective in reducing subjective measures, behavioral responses and toxicant exposure among young adults: A crossover study

**DOI:** 10.1371/journal.pone.0327730

**Published:** 2025-08-27

**Authors:** Natasha Shaukat, Sreshtha Chowdhury, Simanta Roy, Taghrid Asfar, Zoran Bursac, Wasim Maziak

**Affiliations:** 1 Department of Epidemiology, Robert Stempel College of Public Health, Florida International University, Miami, Florida, United States of America; 2 Miller School of Medicine, University of Miami, Miami, Florida, United States of America; 3 Sylvester Comprehensive Cancer Center, University of Miami, Miami, Florida, United States of America; 4 Department of Biostatistics, Robert Stempel College of Public Health, Florida International University, Miami, Florida, United States of America; University College London, UNITED KINGDOM OF GREAT BRITAIN AND NORTHERN IRELAND

## Abstract

**Background:**

This study aims to assess the impact of six evidence-based Waterpipe (WP)-specific pictorial health warnings (HWs) placed on the WP device on puffing behavior, toxicant exposure, subjective smoking experiences, harm perception, motivation and intention to quit among young WP smokers in Florida.

**Methods:**

In a crossover experiment, a total of 100 WP smokers completed two 45-minute ad-libitum WP smoking sessions (without HW vs. with HW on the device) after a 12-hour abstinence. Exhaled Carbon Monoxide (eCO) was measured before and after each session, while puff topography was recorded throughout the smoking session. Additionally, participants completed survey questionnaires before and after the WP smoking sessions to assess subjective smoking experiences, harm perception, motivation to quit, and intention to quit.

**Results:**

Study participants had a mean age of 22.5 years (SD = 3.4), and most (56.6%) were male.The mean e-CO boost (34.16 vs. 26.58) (p = 0.006) was higher in the no-HW condition compared to the HW condition. Differences were also observed between the two conditions for puff topography. For example, median smoking time (46.4 min vs 45.3 min; p < 0.001), median puffing time (4.35 min vs. 4.22 min; 0.004), average puff duration (3.13 sec vs. 2.90 sec; 0.029), and the total number of puffs (95.0 vs. 84.0; 0.039) were lower in the HW compared to no-HW conditions. Subjective measures also demonstrated differences between the two conditions, including greater suppression of the urge to smoke (14.98 vs. 7.51; p < 0.001), reduced puff liking (4.01 vs. 3.63; p = 0.010), and reduced puff satisfaction (4.11 vs. 3.65; p = 0.009) following exposure to HW compared to no-HW conditions. Motivation to quit (p = 0.058), intention to quit (p = 0.659), and harm perception (p = 0.301) increased after smoking the WP with HW; however these were not statistically significant.

**Conclusion:**

Using an evidence-based approach, a large sample size for clinical studies, and an efficient and sensitive within-subject design, this study demonstrates that pictorial HWs on WP devices effectively reduce smoking behavior, toxicant exposure, and subjective experiences while increasing harm perception, intention, and motivation to quit WP smoking.

## Introduction

Waterpipe (WP) smoking has become a global public health concern [1,2]. Evidence shows that WP smoking is prevalent globally, with disproportionately higher rates among youth compared to adults [2,3]. This prevalence is higher among college students and young women [3–6]. In the US, the CDC’s 2018 Monitoring the Future survey found that 12.3% of young adults aged 19–30 had smoked a WP in the past year [7]. Nationally representative data from the Population Assessment of Tobacco and Health (PATH) study showed that -between 2013 and 2018- 18.5% of young adults (aged 18–24) initiated WP smoking, and 14.1% increased their smoking frequency over time [8]. Additionally, recent data from a PATH study assessing WP use from 2013–2021 reported increasing trends of WP smoking among young adults (aged 18–24) as compared to other age groups [9].

Evidence shows that WP smoking can lead to dependence and many known smoking-related diseases [10,11]. Despite these health risks, many WP smokers perceive it as less addictive and harmful than cigarette smoking [12]. These misperceptions can be due to the belief that the water in the device acts as a filter for smoking-related toxicants [13,14], or lack of knowledge about harmful exposures associated with WP smoking [15].Addressing these misconceptions is essential to help respond to the rise of WP smoking and its associated morbidity and mortality [16].

Health communication messaging about the harms of smoking has been among the most successful tobacco control measures worldwide [17]. For example, cigarette health warnings (HWs) have been shown to increase harm perceptions, intention to quit, quitting smoking, as well as prevent smoking initiation [18]. For WP smoking, there is currently a mismatch between the level of evidence about its harmful and addictive properties and public knowledge about them [16]. To correct that, population health communication strategies specific to WP smoking are needed, given the many differences between WP and cigarette smoking (e.g., WP is a multipart device and not as portable as cigarette).

Our team started these efforts several years ago, by conducting a critical appraisal of the literature about the harmful effects of WP smoking, followed by developing evidence-based pictorial HWs [19]. In the last stage, we have been testing the HWs via a variety of modalities (e.g., qualitative research, online experiment, quantitative assessments) and among different populations [20–23]. Our pilot data from testing two HW prototypes on the WP device with 30 participants demonstrated their promise to negatively affect the smoking experience and reduce exposure to toxicants (e.g., CO) [24]. Building on this work, in this study, we expanded the sample size to 100 participants and tested a total of six HWs (the original two plus four additional HWs) to provide a more comprehensive assessment. We also expand on the studied parameters to include important additional outcomes such as withdrawal symptoms, craving, and quit motivation and intention.

This study aimed to test 6 evidence-based pictorial HWs on a variety of WP smokers’ subjective (withdrawl, cravings, puff liking, harm perception, motivation, and intention to quit) and objective outcomes (puff topography, expired Carbon Monoxide) in a within-subject crossover experiment. The tested HWs underwent a comprehensive development process, including feedback from experts and members of the target population (WP smokers) [19]. We hypothesize that HWs on the device will reduce smoking (puffing) intensity, satisfaction, exposure to toxicants, and dependence parameters and increase harm perception and intention to quit compared to no-HW control.

## Methods

### Design

Participants were randomly assigned to one of the 6 HW conditions and underwent 2 WP smoking sessions that differed by HW on the WP device (HW vs. no-HW), with pre-/post-assessments of smoking assessment of puffing behavior, eCO, subjective measures including the Duke Sensory Questionnaire (DSQ), and the Minnesota Nicotine Withdrawal Scale (MNWS). However, additional assessments were included during the second phase of the study, including harm perception and the intention and motivation to quit WP smoking, among a sample of 40 participants.

### Participants and recruitment

Regular WP smokers who smoked WP at least once a week in the past 6 months (18–35 years; n = 100) were recruited from the metropolitan area of Miami, Florida, via flyers, word of mouth, and snowball sampling conducted in two phases: November 2018–March 2019 and December 2022–November 2024. Exclusion criteria included a self-reported history of chronic health problems, psychiatric conditions, regular use of prescription medications (other than vitamins or birth control pills), and current use of > 5 cigarettes or other tobacco/nicotine products in the month preceding the study. Individuals identified as potentially eligible based on the phone screening were asked to attend an onsite screening. Women were excluded if they were breastfeeding or tested positive for pregnancy at the time of screening. This study was approved by the Institutional Review Board of Florida International University and the University of Miami. It was conducted at the clinical research lab for tobacco smoking at the Florida International University.

### Procedures

After screening, eligible participants provided informed consent for the study during the first session. Participants completed two smoking sessions, one using WP without HW and one with WP with one of the six randomly assigned HWs on the device. The HWs were placed on the body of the WP device at participants’ eye level. These were glossy, smooth-to-touch stickers measuring (4.2 inch x 2.5 inch). The no-HW condition always came first to avoid the carryover effect. The sessions were separated by a 48-hour washout period, preceded by > 12 hours of tobacco abstinence, and confirmed by eCO < 6 ppm. Participants were instructed to smoke the same brand and their preferred flavor ad-libitum for up to 45 minutes in both sessions. During each session, participants were seated in a private room with a comfortable reclining chair and were give the choice to use laptop/mobile phones/watch movies while smoking WP. Interactions with researchers were minimal and limited to standardized instructions given before each session; no engagement occurred during the smoking period to avoid influencing participant behavior.

**Fig 1:**
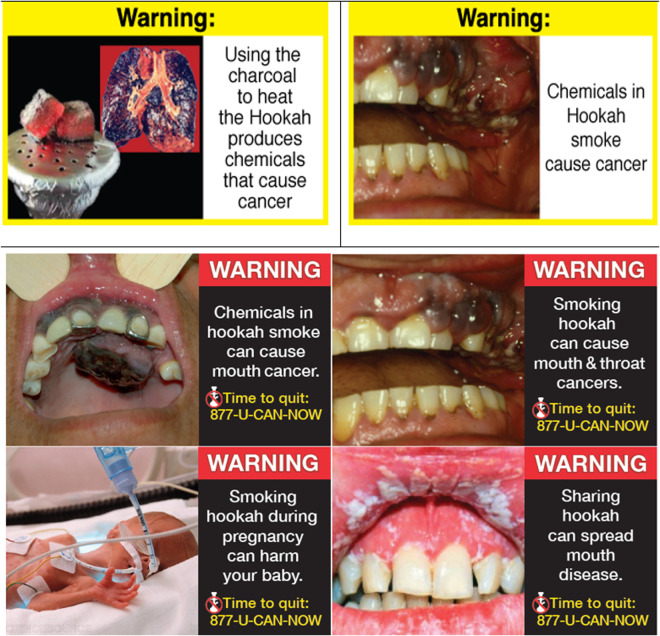
Health warnings.

The study was conducted in two phases, where phase one was a pilot testing of limited outcomes. Based on the pilot’s encouraging results, we expanded the sample size and measured outcomes in phase 2. Thus, the study protocol and assessments for both phases included puff topography, eCO, subjective measures (Duke Sensory Questionnaire (DSQ), and the Minnesota Nicotine Withdrawal Scale (MNWS). In addition, for a subsample of 40 participants (phase 2), we added harm perception, intention, and motivation to quit WP smoking. Similarly, while all HWLs used were evidence -based, the ones added in phase 2 underwent a more comprehensive development process, including feedback from experts and members of the target population (WP smokers) [19]. The health warnings are shown in (Fig 1).

Puff topography was measured throughout all WP smoking sessions, and eCO measurements were collected pre- and post-WP smoking sessions. Subjective responses were assessed in both pre- and post-WP smoking sessions, except the Duke Sensory Questionnaire (DSQ), which was administered post-smoking only.

### Outcome measures

**Puff topography** was assessed using a validated device developed by the American University of Beirut [25]. Briefly, a pressure transducer is integrated into the WP hose, and inhalation-induced pressure changes are amplified, digitized, and sampled at a 1000 Hz rate. The software converts signals to airflow (ml/sec). It integrates the flow data, producing measures of total smoking time, puff duration, inter-puff interval (IPI), total puffing time, the number of puffs, total volume inhaled, and average puff volume.

**Expired carbon monoxide (eCo**) was measured initially at the beginning of the smoking session (abstinence verification) and within 5 min after the 45-minute WP smoking session via e-CO monitor (Vitalagraph, Lenexa, KS). eCO boost was calculated as the difference between post- and pre-session values (post – pre).

#### WP-related measures.

WP-related Measures included: age at first WP smoking experience (years); average number of hookahs (heads/bowls) smoked per month; usual type of WP tobacco smoked (flavored or unflavored); perceived differences in smoke production between flavored and unflavored tobacco (flavored, unflavored, or no difference); average duration of a typical WP smoking session (less than 30 minutes, 30–60 minutes, or more than 60 minutes); shared WP with others (yes/no); and usual smoking place (home, friend’s home, public places such as cafés/restaurants,others).

#### Subjective measures.

Tablet-based questionnaires collected subjective information pre- and post-smoking, except the Duke Sensory Questionnaire (DSQ), which was administered post-smoking only.

**Harm perception** was based on one item, “To what extent are you thinking about the serious health effects of WP smoking?” with response options on a ten-point scale ranging from 1 (not at all) to 10 (a lot) [26].

**Intention and motivation to quit** were assessed by the questions: “Do you intend to quit hookah smoking?” with response options (no, yes in the next month, yes in the next 6 months, and yes, in the future) and “How motivated are you to quit hookah smoking in the next month?” with response options (not motivated, somewhat motivated, and very motivated). For analysis, intention to quit was dichotomized as “yes” (any positive intention) versus “no,” and motivation to quit as “motivated” (somewhat or very motivated) versus “not motivated” [27].

**Duke Sensory Questionnaire (DSQ)** The appeal and subjective effects of the WP post-smoking were evaluated using the Duke Sensory Questionnaire (DSQ) [28], which included nine questions related to puff liking, puff satisfaction, nicotine in puffs, and puff strength on various areas of the body (tongue, nose, mouth, throat, windpipe, and chest), as well as similarity to the participant’s own brand. Participants rated these items using a 7-point Likert scale (1 = ‘not at all,’ 7 = ‘extremely’). DSQ ratings for puff strength on the tongue, nose, mouth and throat, windpipe, and chest were collapsed to form an overall strength score (range 7–35).The DSQ was administered post-WP smoking session [28].

**Minnesota Nicotine Withdrawal Scale (MNWS***)* The Minnesota Nicotine Withdrawal Scale (MNWS) is an 11-item validated questionnaire that assesses nicotine withdrawal and craving symptoms (urges to smoke, irritability/frustration/anger, anxiety, difficulty concentrating, restlessness, hunger, impatience, craving a hookah/nicotine, drowsiness, depression/feeling blue, and desire for sweets). Participants responded to each item on a slider scale by circling any point on the line. Responses ranged from 0 (not at all) to 100 (extremely). An overall score was calculated by averaging all the items [29].

### Data analysis

Baseline descriptive statistics of the participants were summarized as mean (M) and standard deviation (SD), median and interquartile range (IQR), or proportions, as appropriate.The normality of the data was assessed using box plots/Q-Q plots. Based on that, paired sample t-test,Wilcoxon signed-rank test, and Chi-square test were applied as appropriate to examine mean differences and/or distributional equality in puffing parameters, eCO, and subjective measures scores (e.g., MNWS, DSQ, harm perception, motivation, and intention to quit) between smoking conditions (HW vs. no-HW). The level of significance was set at an alpha level of 0.05. All analyses were conducted using SPSS version 25.

## Results

### Demographics

Study participants (n = 100) had a mean age of 22.5 years (SD = 3.4). Most (56.6%) participants were male. The mean age for starting smoking WP was 18 years (SD = 2.0). The median WP smoking frequency was 3 (heads/bowls) per month (Table 1).

**Table 1:** Baseline characteristics for the overall sample (n = 100).

Variables	Overall sample
Age in years, mean (SD)	22.5 (3.42)
Age of first smoking waterpipe, mean (SD)	18 (2.04)
Gender (male) (%)	56.6
Race	
Hispanic (%)	59.6
Non-Hispanic (%)	40.4
Education level	
High School graduate & college (%)	45.9
Associate and above (%)	54.1
Currently Employed (%)	31.3
Waterpipe use/month, median (IQR)	3 (2,5)

### WP smoking topography and expired carbon monoxide (eCO)

During a 45-minute ad-libitum WP smoking session, all topography measures were higher in sessions without HW. Significant differences were found between the two conditions for median smoking time (46.4 vs. 45.3 min; p =<0.001), puffing time (4.35 vs. 4.22 min; p = 0.004), average puff duration (3.13 vs. 2.90 sec; p = 0.029), and number of puffs (95 vs 84; p = 0.039) (Table 2).

**Table 2:** Waterpipe smokers topography measures according to HW and no-HW conditions (n = 99)^a^.

Topography Parameters	No- HW		HW		P-Value
	Median	IQR	Median	IQR	
Smoking time, mins	46.4	44.9-47.7	45.3	34.5-46.8	**< 0.001**
Puffing time, mins	4.35	3.32-4.35	4.22	3.03-5.57	**0.004**
Average puff duration, sec	3.13	2.1-4.0	2.90	2.26-3.66	**0.029**
Average flow rate, ml/sec	13.71	9.49-17.78	12.99	8.79-17.21	0.603
Inter-puff interval, sec	23.00	14.64-34.57	25.05	15.99-34.57	0.870
Number of puffs	95.00	63-145	84.00	62-123	**0.039**
Total Inhaled volume, L	62.57	41.67-103.9	52.94	28.68-89.08	0.696
Average puff volume, L	0.73	0.46-1.04	0.59	0.35-0.95	0.951
Maximum puff volume, L	1.84	1.27-2.55	1.58	1.09-2.05	0.711

^a^One participant was missing data.

Bold indicates significant differences (p < 0.05).

Exposure to eCO differed between the two conditions, with eCO boost being higher in the no-HW condition (34.16 vs. 26.58; p = 0.006). (Table 3).

**Table 3:** Waterpipe smokers eCO levels (n = 99)^b^.

eCO levels	No -HW Mean (SD)	HW Mean (SD)	P-Value
Before the session	1.46(1.03)	1.69(1.22)	0.190
After the session	35.63(38.31)	28.26 (28.41)	**0.009**
eCo Boost (Post-Pre)	34.16 (38.21)	26.58 (28.36)	**0.006**

^b^One participant was missing data.

Bold indicates significant differences (p < 0.05).

### Subjective measures

Subjective measures also showed differences between the two conditions. For the MNWS, pre- and post-smoking assessments were collected, and change scores (post minus pre) were analyzed. WP smokers in the HW condition reported significantly greater suppression of urge to smoke compared to the no-HW condition (7.51 vs. 14.98; p < 0.001) (Table 3). The DSQ was administered post-smoking only. Compared to the no-HW condition, participants reported lower DSQ scores for puff liking (3.63 vs. 4.01; p = 0.010) and puff satisfaction (3.65 vs. 4.11; p = 0.009) during the HW condition. (Table 4).

**Table 4:** Waterpipe smokers subjective responses with and without Health Warnings according to HW and no-HW condition (n = 100).

Scale	Items	No-HW (Post-Session Mean)	HW (Post-Session Mean)	P-value
**Duke Sensory Questionnaire**				
	How much did you like the puffs?	**4.01**	**3.63**	**0.010**
	How satisfying were the puffs?	**4.11**	**3.65**	**0.009**
	How high in nicotine were the puffs?	3.65	3.71	0.661
	How similar to your own brand/flavor were the puffs?	4.04	3.70	0.093
	Overall, Strength Score	17.92	18.20	0.688
	Rate the strength of the puffs on tongue.	3.55	3.69	0.393
	Rate the strength of the puffs on nose.	3.01	3.15	0.363
	Rate the strength of the puffs on the back of mouth/throat.	3.81	3.93	0.494
	Rate the strength of the puffs on the windpipe.	3.82	3.87	0.793
	Rate the strength of the puffs on the chest.	3.68	3.65	0.506
**Minnesota Nicotine Withdrawal Scale (MNWS): (Mean score change, pre-post)**				
	Total Score (MNWS)	9.26	31.09	0.120
	Urges to smoke	**7.51**	**14.98**	**0.002**
	Irritability/frustration/anger	2.80	2.91	0.955
	Anxious	1.39	3.16	0.438
	Difficulty concentrating	−0.54	1.69	0.387
	Restlessness	−1.57	−0.50	0.602
	Hunger	−0.42	−2.34	0.652
	Impatient	0.50	2.75	0.461
	Craving a hookah/nicotine	7.52	10.69	0.272
	Drowsiness	−8.03	−3.22	0.092
	Depression/feeling blue	2.71	0.97	0.330
	Desire for sweets	−0.26	−1.65	0.701

*DSQ items are reported as post-session mean scores. *Higher DSQ scores reflect greater sensory** satisfaction or perception of smoke strength.

MNWS items are reported as mean change scores (post minus pre). Higher MNWS scores reflect greater withdrawal symptom severity.

Bold indicates significant differences (p < 0.05).

**Fig 2:**
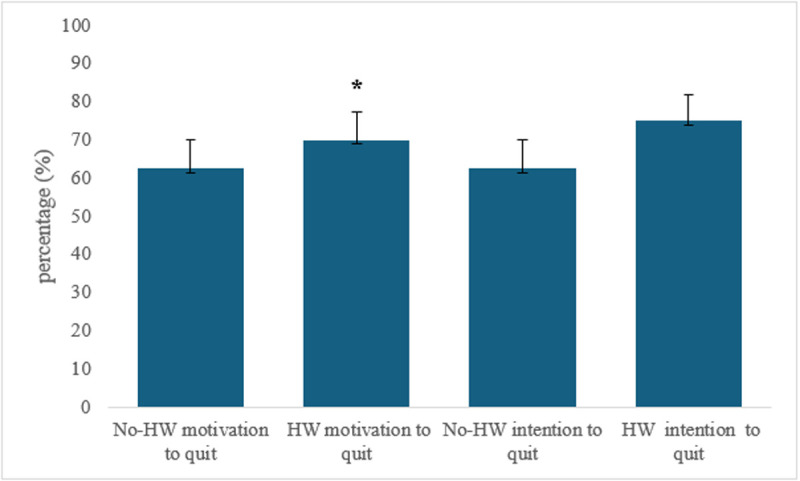
Percentage of WP smokers post-session intention and motivation to quit WP smoking (n = 40). * Indicates significant differences (p < 0.05).

**Fig 3:**
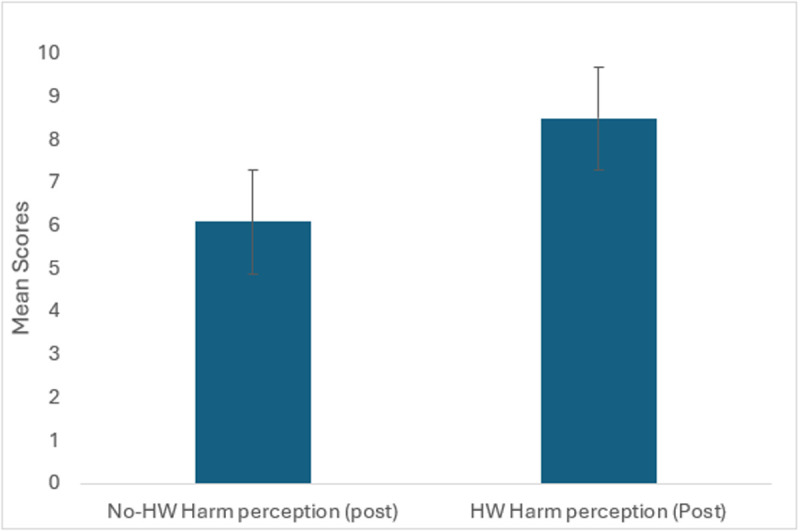
Mean scores for post-session harm perception responses among WP smokers (n = 40).

Figs 2 and 3 depict post-session responses for motivation to quit WP smoking, intention to quit WP smoking, and WP harm perception for the two conditions, respectively. Post-session intention to quit was dichotomized as ‘Yes’ (‘Yes, in the next month,’ ‘Yes, in the next 6 months,’ or ‘Yes, in the future’) versus ‘No’ (‘No’) and compared between HW and no-HW conditions. Post-session motivation to quit was dichotomized as ‘Yes’ (‘Somewhat motivated’ or ‘Very motivated’) versus ‘No’ (‘Not motivated’) and compared between HW and no-HW conditions. About 70% of participants in the HW condition reported higher post-session motivation to quit WP smoking (p = 0.058) compared to 62.5% in the no-HW condition. Similarly, although a greater proportion of participants in the HW condition (75%) expressed a post-session intention to quit compared to the no-HW condition (62.5%), this difference was not statistically significant (p = 0.659) (Fig 2).

Post-session perceived harm mean scores were also higher in the HW condition (8.49) compared to the no-HW condition (6.09), but this difference was not statistically significant (p = 0.301) (Fig 3).

## Discussion

The data suggest that exposure to HW on WP devices resulted in less intensive smoking/puffing behavior, exposure to toxicants, and subjective experiences. However, it increased harm perception, motivation to quit, suppression of withdrawal and craving, and motivation to quit waterpipe. These results provide comprehensive and consistent support for the value of implementing HW regulations on the WP device in curtailing WP smoking experiences, addiction, and exposure to toxicants [17,23,24,30].

The observed reduced puffing patterns related to exposure to the HW included shorter sessions, fewer puffs, and reduced puffing duration. Given the strong correlation between puffing patterns and CO production [31–33], these reduced puffing patterns likely underlie the reduction in exposure to CO observed in the HW condition. While we measured only CO in this study, the reduction documented in the HW condition will likely affect many of the other inhaled toxicants associated with WP smoking. In contrast to cigarettes, most of the toxicants in WP smoke originate from charcoal combustion rather than heated tobacco [7,34]. These include carbon monoxide, PAHs, nicotine, and aldehydes, which are implicated in a variety of smoking-related cardiovascular diseases, respiratory illnesses, and cancer [10,11].

Our study shows that exposure to HW led to negative subjective experiences (e.g., puff liking, satisfaction) and greater suppression in withdrawal and craving symptoms. Negative emotions triggered by the HW are likely responsible for making the smoking experience less enjoyable and desirable [35], leading to worse performance on abstinence-induced craving and withdrawal. Repeated exposure to such HWs therefore, can potentially attenuate nicotine dependence among WP smokers. In tandem with these, HW led to increased harm perception, motivation, and intentions to quit the WP. Studies show a strong association between harm perception not only with quitting but also with initiation [17,30], placing HW as a promising strategy to affect multiple junctions in the WP epidemic.

### Strengths and limitations

Our study had several strengths, including the within-subject design, 12-hour abstinence (verified through eCO) before each session, and 48-hour washout period between sessions. On the limitations side,first, the laboratory environment is inevitably different from the typical WP lounge vibe. To minimize this effect, we equipped our lab with a reclining chair, allowed participants to use laptop/moblie phones/watch movies of their choice, during sessions. These features made the session area more relaxing and entertaining to facilitate a natural smoking experience. Since the lab conditions were similar for all sessions and only the exposure of interest (HW) was manipulated, the documented differences likely represent real responses to the health warning. Another limitation is that for some outcomes like harm perception and intention to quit, the results were not statistically significant, likely because they were assessed only in a subsample of 40. However, our results in terms of the subjective effects of HW are comprehensive and consistent with earlier studies showing a great correlation between harm perception and intention to quit [17,30]. Similarly, the 6 HW used in this study did undergo different processes of selection with the latter one being more thoroughly developed before use. However, all the HWs were evidence-based, and sensitivity analysis did not show meaningful differences between the performance of these HWs on main study outcomes. Furthermore, the fixed order of sessions (HWL condition comes last) was mandated by the expected lasting carryover effect of graphic HWLs, which would bias the assessment if used first, not counterbalancing may have introduced systematic differences (order effect). However, these are likely to be minimal, given our young sample and nonmedical intervention. Finally, our study is an acute model study and needs to be supplemented in the future by longer exposures to HW and assessments of its effect on important long-term outcomes such as quitting WP smoking.

## Conclusions

Using an evidence-based approach, a large sample size for clinical studies, and an efficient and sensitive within-subject design, this study provides credible evidence that pictorial HWs on WP devices are effective in reducing WP smokers’ behavior, toxicant exposure, and subjective experiences while increasing harm perception and motivation to quit. These results provide strong support for considering HWs as an integral part of public health strategies aimed at reducing WP smoking and its related morbidity. Our study findings can guide policymakers to improve current WP HWs by (1) including pictorials, (2) addressing harm of WP smoking beyond nicotine (e.g., charcoal), and (3) placing warnings directly on the waterpipe device, not just the packaging, to improve visibility and impact.Future research can focus on understanding the long-term effects of exposure to HWs on WP smoking behavior, as well as testing HWs in real-world popular WP smoking settings, such as hookah cafés and bars.

## Supporting information

S1 Appendix Dataset. (XLSX)

S2 Appendix Questionnaire. (PDF)
