# Supplementary material for: Waterpipe-specific pictorial health warning labels are effective in reducing subjective measures, behavioral responses and toxicant exposure among young adults: A crossover study
Source: PLoS One. 2025 Aug 27;20(8):e0327730. doi: 10.1371/journal.pone.0327730 (PMC12385414; doi:10.1371/journal.pone.0327730)
Supplement: S2 Appendix — (PDF) [file pone.0327730.s002.pdf]

Understanding Health warning effects on waterpipe smokers' harm perception, intention to quit, smoking experiences and exposures to toxins

Baseline Questionnaire – Screening Questions to Determine Eligibility

Participant ID: ________________

1. What is your date of birth? _______________

2. What is your age? _____________

3. What identifier best describes you?

   □ Male

   □ Female

   □ Other

   3a. If female, ask the following: Are you currently pregnant or breastfeeding?

      □ Yes

      □ No (confirmed by urine analysis)

4. Have you ever smoked cigarettes in the past year?

   □ Yes

   □ No

   4a. If yes, ask the following: Which statement best describes your cigarettes smoking in the past year?

      □ 1-5 cigarettes/month

      □ 6-10 cigarettes/month

      □ 11-20 cigarettes/month

      □ > 20 cigarettes/month

5. Do you smoke hookah?

□ Yes

□ No

5a. If yes, ask the following: When did you start smoking hookah?

□ Within the past 6 months

□ More than 6 months ago but less than one year

□ More than 1 year ago

6. Which statement best describes your hookah smoking in the past 6 months

□ Smoke hookah less than once a week

□ Smoke hookah at least once a week, but not everyday

□ Smoke hookah every day or on most days

□ I did not smoke hookah in the past 6 months

7. Do you regularly use any of the following tobacco/nicotine products?

□ E-cigarettes

□ Cigars

□ Cigarillos/Little cigars

□ Smokeless tobacco

□ Other [please specify] _____________________

□ None

7a. If yes, ask the following: Which statement best describes your tobacco use in the past year?

□ Not applicable

□ 1-5 times/month

□ 6-10 times/month

□ > 10 times/month

8. Do you have any chronic health concerns or problems?

□ Yes

□ No

8a. If yes, ask the following: Please describe the concern or problem: ____

9. Are you under a doctor's care for a medical condition?

□ Yes

□ No

9a. If yes, ask the following: Please describe the condition____

10. Are you taking any prescription or over-the-counter medications?

□ Yes (other than birth control or vitamins)

□ No

10a. If yes, ask the following: Please identify the medications(s) ____

11. Do you have any psychiatric conditions such as depression or anxiety?

□ Yes

□ No

11a. If yes, ask the following: Please describe the condition ____

12. Have you ever been diagnosed with high or low blood pressure?

□ Yes

□ No

13. Do you give permission for your data to be stored and used for research related to future studies, or to determine if you are eligible for future studies?

□ Yes

□ No

Sociodemographic Information

1. Sex

□ Male

□ Female

□ Other

2. How old are you?

__________ Years-old

3. Are you Hispanic, [Latino or Latina], or of Spanish origin? Choose all that apply.

□ No, not of Hispanic, [Latino or Latina], or Spanish origin

□ Yes, Mexican, Mexican American, [Chicano or Chicana]

□ Yes, Puerto Rican

□ Yes, Cuban

□ Yes, Another Hispanic, [Latino or Latina], or Spanish origin

□ Don't know

4. What is your race? Choose all that apply

□ White

□ Black or African American

□ American Indian or Alaska Native

□ Asian

□ Chinese

□ Pacific Islander

□ Other

5. What is the highest degree or level of school you have completed? *If currently enrolled, highest degree received.*

   □ No schooling completed

   □ Some high school, no diploma

   □ High school graduate, diploma or the equivalent (for example: GED)

   □ Some college credit, no degree

   □ Associate degree

   □ Bachelor's degree

   □ Master's degree

   □ Doctorate degree

6. Employment Status: Are you currently…?

   □ Employed for wages

   □ Self-employed

   □ Out of work and looking for work

   □ Out of work but not currently looking for work

   □ A homemaker

   □ A student

   □ Military

   □ Retired

   □ Unable to work

Water Pipe Use

1. At what age did you smoke hookah for the first time in your life?

_____ Years

2. On average how many hookahs (heads/bowels) you usually smoke per month?

_____ hookah/months

3. What type of hookah tobacco do you usually smoke?

   □ Flavored tobacco (___________________ please specify type)

   □ Unflavored

4. Which hookah tobacco produced greater amount of smoke during your smoking sessions?

   □ Flavored tobacco

   □ Unflavored tobacco

   □ No difference

5. What is the average time you usually spend during a hookah smoking session?

   □ Less than 30 minutes

   □ 30 – 60 minutes

   □ More than 60 minutes

6. Do you usually share the same hookah with others?

   □ Yes

   □ No

7. Where do you usually smoke hookah?

   □ At home

   □ At a friend's

   □ In public places (e.g., a café/restaurant)

   □ Other places

Pre – Session Questionnaire

Minnesota Nicotine Withdrawal Scale (MNWS)

Instructions: Participants respond to each item by circling any point on the line. Response is measured from 0 (not at all) to 100 (extremely).

1. Urges to smoke	0 10 20 30 40 50 60 70 80 90 100
2. Irritability/frustration/anger	0 10 20 30 40 50 60 70 80 90 100
3. Anxious	0 10 20 30 40 50 60 70 80 90 100
4. Difficulty concentrating	0 10 20 30 40 50 60 70 80 90 100
5. Restlessness	0 10 20 30 40 50 60 70 80 90 100
6. Hunger	0 10 20 30 40 50 60 70 80 90 100
7. Impatient	0 10 20 30 40 50 60 70 80 90 100
8. Craving a hookah/nicotine	0 10 20 30 40 50 60 70 80 90 100
9. Drowsiness	0 10 20 30 40 50 60 70 80 90 100
10. Depression/feeling blue	0 10 20 30 40 50 60 70 80 90 100
11. Desire for sweets	0 10 20 30 40 50 60 70 80 90 100

Intention and motivation to Quit Smoking

1. Do you intend to quit hookah smoking?
   □ No
   □ Yes, in the next month
   □ Yes, in the next 6 months
   □ Yes, in the future

2. How motivated are you to quit hookah smoking in the next month?

   □ Not motivated

   □ Somewhat motivated

   □ Very motivated

Harm Perception

Instructions: Please respond to the following statement and questions by marking the appropriate number on the line.

1. To what extent are you thinking about the serious health effects of hookah smoking?

   Not at all A lot

   |------------|------------|------------|------------|------------|-------------|-------------|-----------|-----------|-------------|

   0 1 2 3 4 5 6 7 8 9 10

Post – Session Questionnaire

Minnesota Nicotine Withdrawal Scale (MNWS)

Instructions: Participants respond to each item by circling any point on the line. Response is measured from 0 (not at all) to 100 (extremely).

1. Urges to smoke	0 10 20 30 40 50 60 70 80 90 100
2. Irritability/frustration/anger	0 10 20 30 40 50 60 70 80 90 100
3. Anxious	0 10 20 30 40 50 60 70 80 90 100
4. Difficulty concentrating	0 10 20 30 40 50 60 70 80 90 100
5. Restlessness	0 10 20 30 40 50 60 70 80 90 100
6. Hunger	0 10 20 30 40 50 60 70 80 90 100
7. Impatient	0 10 20 30 40 50 60 70 80 90 100
8. Craving a hookah/nicotine	0 10 20 30 40 50 60 70 80 90 100
9. Drowsiness	0 10 20 30 40 50 60 70 80 90 100
10. Depression/feeling blue	0 10 20 30 40 50 60 70 80 90 100
11. Desire for sweets	0 10 20 30 40 50 60 70 80 90 100

Duke Sensory Questionnaire (DSQ)

Instructions: Please evaluate the hookah you just smoked by marking the appropriate area for each of the following questions.

	Not at all	Very Little	A little	Moderately	A lot	Quite a lot	Extremely
1. How much did you like the puffs?							
2. How satisfying were the puffs?							
3. How high in nicotine were the puffs?							
4. How similar to your own brand/flavor were the puffs?							
5. Rate the strength of the puffs on tongue.							
6. Rate the strength of the puffs on nose.							
7. Rate the strength of the puffs on the back of mouth/throat.							
8. Rate the strength of the puffs on the windpipe.							
9. Rate the strength of the puffs on the chest.							

Intention and motivation to Quit Smoking

1. Do you intend to quit hookah smoking?
   □ No
   □ Yes, in the next month
   □ Yes, in the next 6 months
   □ Yes, in the future

2. How motivated are you to quit hookah smoking in the next month?

   □ Not motivated

   □ Somewhat motivated

   □ Very motivated

3. How interested are you in using the tobacco you just smoked again in the future? Response is measured from 0 (not at all) to 100 (extremely)

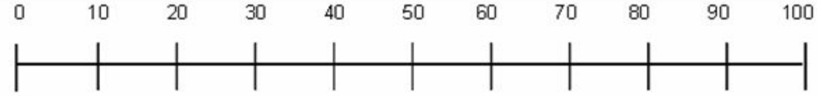

4. How likely would you use the tobacco you just smoked if it was the only product available in the market? Response is measured from 0 (not at all) to 100 (extremely).

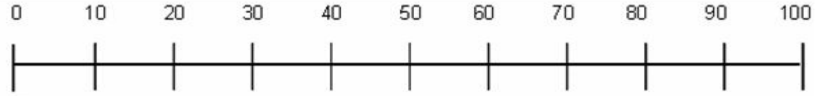

Harm Perception

Instructions: Please respond to the following statement and questions by marking the appropriate number on the line.

1. To what extent are you thinking about the serious health effects of hookah smoking?

Not at all A lot

|-------------|-------------|-------------|-------------|-------------|--------------|--------------|------------|------------|-------------|

0 1 2 3 4 5 6 7 8 9 10
